# The influence of soft-tissue volume grafting on the maintenance of peri-implant tissue health and stability

**DOI:** 10.1186/s40729-021-00295-1

**Published:** 2021-02-23

**Authors:** Karina Obreja, Ausra Ramanauskaite, Amira Begic, Maria Elisa Galarraga-Vinueza, Puria Parvini, Frank Schwarz

**Affiliations:** 1 Department of Oral Surgery and Implantology, Johann Wolfgang Goethe-University Frankfurt, Carolinum, Theodor-Stern-Kai 7; Building 29, 60596 Frankfurt, Germany; 2 Federal University of Santa Catarina (UFSC), SC Florianópolis, Brazil

**Keywords:** Soft-tissue grafting, Peri-implant diseases, Dental implants

## Abstract

**Background:**

To investigate the influence of soft-tissue volume grafting employing autogenous connective tissue graft (CTG) simultaneous to implant placement on peri-implant tissue health and stability.

**Material and methods:**

This cross-sectional observational study enrolled 19 patients (*n* = 29 implants) having dental implants placed with simultaneous soft-tissue volume grafting using CTG (test), and 36 selected controls (*n* = 55 implants) matched for age and years in function, who underwent conventional implant therapy (i.e., without soft-tissue volume grafting). Clinical outcomes (i.e., plaque index (PI), bleeding on probing (BOP), probing depth (PD), and mucosal recession (MR)) and frequency of peri-implant diseases were evaluated in both groups after a mean follow-up period of 6.15 ± 4.63 years.

**Results:**

Significant differences between test and control groups at the patient level were noted for median BOP (0.0 vs. 25.0%; *p* = 0.023) and PD scores (2.33 vs. 2.83 mm; *p* = 0.001), respectively. The prevalence of peri-implant mucositis and peri-implantitis amounted to 42.1% and 5.3% in the test and to 52.8% and 13.9% in the control group, respectively.

**Conclusion:**

Simultaneous soft-tissue grafting using CTG had a beneficial effect on the maintenance of peri-implant health.

## Introduction

A major goal of implant therapy is to ensure long-term peri-implant tissue health and create appealing esthetics. To obtain these therapeutic endpoints, soft-tissue grafting procedures performed either simultaneously with or after implant placement have become an indispensable part of contemporary implant dentistry [1].

From a biological point of view, a lack of or reduced height (< 2 mm) of keratinized mucosa (KM) around the implants was shown to jeopardize self-performed oral hygiene measures, which subsequently increased the likelihood of soft-tissue inflammation [1, 2]. As a consequence, soft-tissue grafting procedures aimed at increasing keratinized tissue have been shown to markedly improve peri-implant soft-tissue inflammatory conditions and were associated with higher marginal bone levels compared to the control sites [3]. Moreover, from an esthetic perspective, the presence of KM > 2 mm was demonstrated to be a preventive measure for the occurrence of peri-implant soft-tissue dehiscences [4].

Changes in peri-implant soft-tissue height, particularly on the facial aspect, are a critical factor that may compromise the overall esthetic result of implant-supported restoration [5]. A thin mucosa (also known as a soft-tissue biotype) at the time of implant installation was found to be a crucial component that correlated with facial soft-tissue recession [6–8]. In fact, to attenuate the undesirable changes of the soft-tissue margin, soft-tissue volume augmentation at the time of implant placement was also suggested as a preventive measure [9, 10]. On the contrary, currently available data evaluating procedures to increase mucosal thickness did not show any significant effects on bleeding scores, but higher interproximal marginal bone levels over time when compared with control sites [1]. Due to a lack of reporting, an evaluation of the prevalence of peri-implant disease was not feasible [1].

Therefore, the aim of the present cross-sectional analysis was to assess the influence of soft tissue volume grafting on the peri-implant tissue health and stability.

## Materials and methods

The present investigation was designed as an observational, cross-sectional case–control study evaluating the clinical treatment outcomes of implants inserted simultaneously with (test group) and without (control group) soft-tissue volume augmentation. All patients had received the same implant brand (Ankylos®, Dentsply Sirona Implants, Hanau, Germany) in a single university clinic (Department of Oral Surgery and Implantology, Goethe University, Frankfurt) and were recruited during their yearly maintenance visits.

Patients were included in the study once they were informed about the investigation procedures and gave their written informed consent. The procedures in the present study were in accordance with the Declaration of Helsinki, as revised in 2013, and the study protocol was approved by the local ethics committee (registration number: 78/18).

### Patient selection criteria

The following inclusion criteria were applied for patient selection:

- Patients with > 18 years of age rehabilitated with at least one Ankylos® implant;

- Patients with treated chronic periodontitis and proper periodontal maintenance care;

- Non-smokers, smokers and former smokers;

- A good level of oral hygiene as evidenced by a plaque index (PI) < 1 at the implant level; and

- Attendance of yearly routine implant maintenance appointment.

Patients were excluded for the following conditions: the presence of combined endodontic–periodontal lesions; systemic diseases that could influence the outcome of the therapy, such as diabetes (HbA1c > 7), osteoporosis and antiresorptive therapy; a history of malignancy, radiotherapy, chemotherapy, or immunodeficiency; and pregnancy or lactation at the last follow-up.

### Surgical protocol

**Fig. 1:**
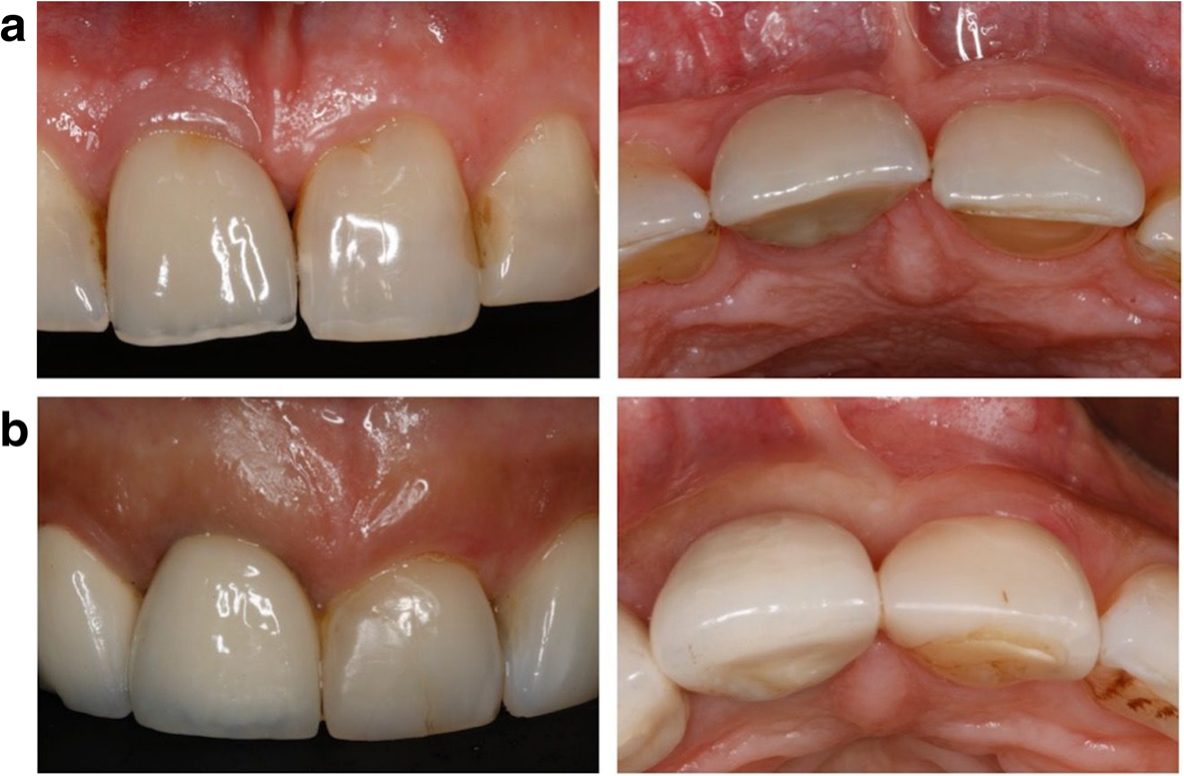
**a** Clinical view of implant 011 at 6 years of healing (control group). **b** Clinical view of implant 011 5 years following soft-tissue volume grafting (test group)

Soft-tissue biotype was assessed preoperatively based on the probe’s transparency at the mid-facial aspect and categorized as thin when the probe was visible and thin when it was not visible. Two-piece platform-switched implants were placed 2–3 mm subcrestally according to the manufacturer’s surgical protocol. Implants in the control group exhibited a thick soft-tissue biotype and therefore underwent a conventional placement protocol (i.e., without soft-tissue volume grafting; Fig. 1a).

**Fig. 2:**
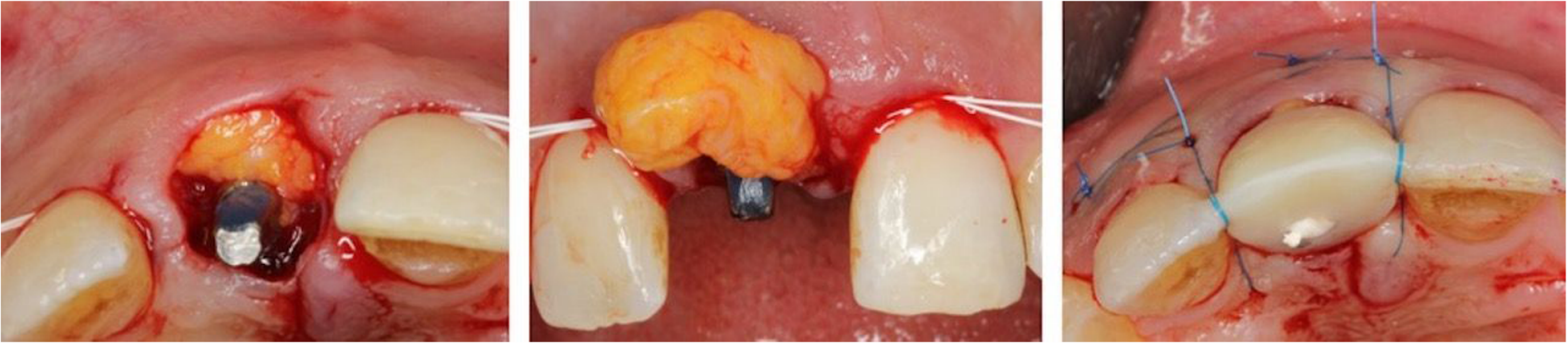
Clinical illustration of soft-tissue grafting procedure. **a**, **b** Connective tissue graft harvested from the hard palate was positioned on the facial aspect via tunneling technique at implant 011. **c** Occlusal view of the surgical site

Implants in the test group presented with a thin soft-tissue biotype, and therefore, a connective tissue graft (CTG) harvested from the hard palate was simultaneously applied on the facial aspect via tunneling technique (Fig. 1b and Fig. 2). All surgeries were performed by one experienced oral surgeon (PP).

### Implant and implant-site characteristics

The following study variables were assessed for the test and control implant sites: (1) implant age (i.e., defined as time after implant placement), (2) implant location in the upper jaw, and (3) implant diameter.

### Clinical measurements

The following clinical parameters were registered at each implant site using a periodontal probe: (1) plaque index (PI) (Löe et al., 1967); (2) bleeding on probing (BOP)—measured as presence/absence; (3) probing depth (PD)—measured from the mucosal margin to the probable pocket; (4) mucosal recession (MR)—measured from the restoration margin to the mucosal margin; and (5) keratinized mucosa (KM) (mm)—measured on the buccal aspects of the implants.

PI, BOP, PD, and MR measurements were performed at six aspects per implant site: mesiobuccal (mb), midbuccal (b), distobuccal (db), mesiooral (mo), midoral (o), and distooral (do). KM measurement was performed at three aspects per implant site: mesiobuccal (mb), midbuccal (b), and distobuccal (db).

The presence of peri-implant diseases at each implant site was assessed as follows [11]:

- Peri-implant mucositis defined as the presence of BOP and/or suppuration with on gentle probing with or without increased PDs compared to previous examinations and an absence of bone loss beyond crestal bone level changes resulting from initial bone remodeling.
- Peri-implantitis defined as the presence of BOP and/or suppuration on gentle probing, increased PDs compared to previous examination, and the presence of bone loss beyond crestal bone level changes resulting from initial bone remodeling.

Radiographs (i.e., panoramic) were just taken when clinical signs suggested the presence of peri-implant tissue inflammation (i.e., the presence of BOP). To estimate the bone level changes at the respective implant sites, these radiographs were compared with those taken following the placement of the final prosthetic reconstruction (i.e., baseline).

### Investigators meeting and calibration

Prior to the start of the study, a calibration meeting was held with each examiner (KO, AB, AR) to standardize (pseudonymous) data acquisition and the assessment of study variables. For the calibration of the examiners, double measurements were performed with a 5-min interval of the assessed clinical parameters in 5 patients with a total of 15 implants. The calibration was acceptable when repeated measurements were similar > 95% level. The documentation of demographic study variables, implant sites’ characteristics, and clinical measurements were documented using a generated standardized data extraction template.

### Statistical analysis

The statistical analysis was performed using a commercially available software program (SPSS Statistics 27.0: IBM Corp., Ehningen, Germany). Descriptive statistics (means, standard deviations, medians and 95% confidence intervals) were calculated for mPI, BOP, PD, and MR values. The analysis was performed at the patient and implant levels. The data were tested for normality by means of the Shapiro-Wilk test. Comparisons of clinical parameters between the test and control groups were performed by employing the Mann-Whitney *U* test. Linear regression analyses were used to depict the relationship between mean BOP, PD, and MR values and KM scores. The alpha error was set at 0.05.

## Results

### Patient and implant sites’ characteristics

The test group included 19 patients (13 women and 6 men) with a total of 29 implants, whereas the control group included 36 patients (20 women and 16 men) with a total of 55 implants. Mean patient age in the test and control groups was 46.24 ± 18.48 and 62.21 ± 14.41 years, respectively. The mean implant functioning time was 4.16 ± 2.06 years for the test group and 7.19 ± 5.25 years for the control group. All implants in the test group revealed a diameter of 3.5 mm with an equal distribution between all regions investigated. In the control group, the most frequent diameter was also 3.5 mm (85.5%), with a predominant implant location in the region of the lateral and central incisors (Table 1).

**Table 1:** Patient and implant site characteristics

	Control group (*n* = 55 implants)	Test group (*n* = 29 implants)
Patient number	*n* = 36	*n* = 19
Patient age (years)	62.21 ± 14.41	46.24 ± 18.48
Patient gender (female/male) (*n*)	20/16	13/6
Implant age years (mean ± SD) (years)	7.19 ± 5.25	4.16 ± 2.06
Region upper jaw		
Premolars/canine/incisives (*n*)	20/15/20	1/4/25
Premolars/canine/incisives (%)	36.4/27.2/33.4	3.4/10.3/86.2
Implant diameter, 3.5/4.5 (*n*)	47/8	29/0
Implant diameter, 3.5/4.5 (%)	85.5/ 14.5	100/ 0

### Clinical measurements

The results of the clinical measurements are presented in Table 2. In general, test and control groups were commonly characterized by low median PI scores at both patient (0.00 vs. 0.21; *p* = 0.093) and implant levels (0.17 vs. 0.17), respectively.

**Table 2:** Clinical parameters (mean ± SD, median and 95% CI)

Clinical parameters	Control group			Test group			***p***
	Mean ± SD	Median	95% CI	Mean ± SD	Median	95% CI	
**Plaque index**							
Patient-level	0.33 ± 0.36	0.21	0.21–0.45	0.18 ± 0.28	0.00	0.04–0.31	0.093
Implant-level	0.35 ± 0.38	0.17	0.25–0.46	0.23 ± 0.32	0.17	0.11–0.35	
**Bleeding on probing (%)**							
Patient-level	28.42 ± 28.35	25.0	18.82–38.01	11.16 ± 14.63	0.00	4.11–18.21	0.023
Implant-level	29.44 ± 30.0	17.0	21.30–37.57	13.83 ± 19.37	0.00	6.46–21.20	
**Probing depth (mm)**							
Patient-level	2.98 ± 0.65	2.83	2.76–3.20	2.36 ± 0.53	2.33	2.10–2.61	0.001
Implant-level	2.99 ± 0.64	2.83	2.81–3.16	2.40 ± 0.54	2.33	2.19–2.61	
**Mucosal recession (mm)**							
Patient-level	0.07 ± 0.19	0	0.00–0.13	0.07 ± 0.18	0	− 0.02 to 0.15	0.76
Implant-level	0.07 ± 0.25	0	0.09–0.14	0.09 ± 0.21	0	0.04–0.17	

Marked differences between test and control groups were noted for median BOP scores, reaching statistical significance at the patient level (0.0 vs. 25.0%; *p* = 0.023).

Similarly, the test group was associated with markedly lower median PD values at both patient (2.33 vs. 2.83 mm; *p* = 0.001) and implant levels (2.33 vs. 2.83 mm), respectively.

Both groups revealed comparable median MR values at both patient (0.0 vs. 0.0 mm; *p* = 0.76) and implant levels (0.0 vs. 0.0 mm), respectively (Table 2).

### Prevalence of peri-implant diseases

The frequency distribution of peri-implant diseases in the test and control groups at patient and implant levels is summarized in Tables 3 and 4.

**Table 3:** Prevalence of peri-implant disease (patient level)

	Control group	%	Test group	%
Healthy	12	33.3	10	52.6
Peri-implant mucositis	19	52.8	8	42.1
Peri-implantitis	5	13.9	1	5.3

**Table 4:** Prevalence of peri-implant disease (implant level)

	Control group	%	Test group	%
Healthy	21	38.2	15	51.7
Peri-implant mucositis	29	52.7	13	44.8
Peri-implantitis	5	9.1	1	3.4

According to the given case definitions, 66.7% of the patients in the control group and 47.4% of the patients in the test group were diagnosed with peri-implant diseases. In the test group, the prevalence of peri-implant mucositis and peri-implantitis amounted to 42.1% and 5.3%. In the control group, the corresponding values were 52.8% and 13.9%, respectively (Table 3).

At the implant level, the prevalence of peri-implant mucositis and peri-implantitis amounted to 44.8% and 3.4% in the test group, and 52.7 and 9.1% in the control group, respectively (Table 4).

### Regression analysis

Cross-tables depicting selected independent variables (PD, MR, and BOP values) and local factors (i.e., KM and Implant age) in both test and control groups are summarized in Tables 5 and 6.

**Table 5:** Test group (*n* = 29 implants). Cross-tables of BOP/PD/MR values and (1) KM and (2) implant age (months)

**1) BOP values**	**KM**		
	< 2 mm	≥ 2 mm	
**0**	0	15	
**< 33%**	0	8	
**< 67%**	0	5	
**> 67%**	0	1	
	**Implant age**		
	1–24	24–60	> 60
**0**	2	7	6
**< 33%**	2	3	3
**< 67%**	0	3	2
**> 67%**	0	1	0
**2) PD values**	**KM**		
	< 2 mm	≥ 2 mm	
**1–3 mm**	0	22	
**4–6 mm**	0	7	
**> 7 mm**	0	0	
	**Implant age**		
	1–24	24–60	> 60
**1–3 mm**	3	11	8
**4–6 mm**	1	3	3
**> 7 mm**	0	0	0
**3) MR**	**KM**		
	< 2 mm	≥ 2 mm	
**0 mm**	0	24	
**> 0 mm**	0	5	
	**Implant age**		
	1–24	24–60	> 60
**0 mm**	4	11	9
**> 0 mm**	0	3	2

**Table 6:** Control group (*n* = 55 implants). Cross-tables of BOP/PD/MR values and (1) KM and (2) implant age (months)

**1) BOP values**	**KM**		
	< 2 mm	≥ 2 mm	
**0**	1	20	
**< 33%**	0	8	
**< 67%**	2	12	
**> 67%**	2	10	
	**Implant age**		
	1–24	24–60	> 60
**0**	6	5	10
**< 33%**	1	5	2
**< 67%**	0	4	10
**> 67%**	0	4	8
**2) PD values**	**KM**		
	< 2 mm	≥ 2 mm	
**1–3 mm**	2	38	
**4–6 mm**	3	12	
**> 7 mm**	0	0	
	**Implant age**		
	1–24	24–60	> 60
**1–3 mm**	7	10	23
**4–6 mm**	0	8	7
**> 7 mm**	0	0	0
**3) MR**	**KM**		
	< 2 mm	≥ 2 mm	
**0 mm**	2	46	
**> 0 mm**	3	4	
	**Implant age**		
	1–24	24–60	> 60
**0 mm**	7	16	25
**> 0 mm**	0	2	5

In the test group, the linear regression analysis failed to reveal any significant correlations between KM and the independent variables investigated.

In the control group, a significant correlation was noted between KM and MR values (*R*^2^ = 0.155; *B* = − 0.072; *p* = 0.003) (Tables 5 and 6).

## Discussion

The present cross-sectional analysis aimed at investigating the influence of soft-tissue volume grafting employing autogenous CTG simultaneous to implant placement on peri-implant tissue health and stability. Based on the clinical parameters investigated, it was noted that the patients in the test group revealed significantly lower BOP and PD scores when compared with those of the control group. This was associated with a lower prevalence of peri-implant diseases, particularly of patients diagnosed for peri-implantitis. In this context, it must be emphasized that the latter assessment was based on recently established case definitions and considered previous examination data [11].

Basically, the present results do not confirm the findings of a recent systematic review and meta-analysis, since soft tissue grafting procedures by means of CTG were not associated with any significant differences in BOP or PD values as compared to control treatments [1]. The analysis was based on a total of 6 randomized (*n* = 2)/controlled clinical (*n* = 4) studies reporting on a total of 260 systemically and periodontally healthy patients over a mean follow-up period of 57 months [9, 12–16]. Except for one study [17], the implants were placed immediately and soft tissue grafting was accomplished either at implant placement [9, 12, 15], or after a healing period of 3 months [13, 14, 16]. At test sites, the range of mean BOP values was 20–35% at baseline and amounted to 20–56% at follow-up [14–17]. The corresponding values at control sites were 21–40% at baseline and 33–46% at follow-up [1]. A total of five studies [9, 13–16] failed to identify any significant effects of soft-tissue volume grafting on mean PD values. In particular, at test sites, the range of mean PD values was 2.50–3.45 mm at baseline and amounted to 3.67–4.09 mm at follow-up. At control sites, these values were 2.50–3.20 mm at baseline and 3.20–3.97 mm at follow-up [1]. One study focusing on immediate implant placement with simultaneous soft-tissue volume grafting reported on significantly lower PD values at test sites when compared with control sites [12].

The meta-analysis failed to reveal any significant differences in either plaque, BOP, or PD scores (i.e., changes or endpoint values) between test and control groups. However, significantly less marginal bone loss over time was observed with the use of CTG [*n* = 2; WMD = 0.110; 95% CI (0.067; 0.154); *p* < 0.001] when compared to sites without grafting [1].

The discrepancy noted between the present analysis and the aforementioned systematic review may, at least in part, be explained by the fact that the included studies [9, 12–16] did not consider BOP or PD as primary outcomes measures. Accordingly, the power of these studies may not have been sufficient to rule out potential differences between groups. Moreover, it needs to be emphasized that none of the evaluated studies [9, 12–16] used case definitions for the evaluation of the occurrence of peri-implant diseases [1].

The present study did not consider to routinely take radiographs during follow-up, but just limited the indication to those patients exhibiting clinical signs of peri-implant tissue inflammation [18]. Accordingly, the influence of soft-tissue volume grafting procedures on marginal bone level changes could not be assessed.

When further evaluating the present data, it was also noted that, in contrast to implants of the test group, control sites revealed a significant correlation between KM and MR values. In this context, it must be emphasized that a major drawback of the present study was the lack of a quantification of the horizontal mucosal thickness (i.e., biotype) during follow-up. That was due to the fact that the assessment of the biotype is challenging at diseased implant sites, since the inflammatory lesion is inevitably associated with an increase in mucosal thickness [19]. As a consequence of the notable prevalence of peri-implant diseases in both groups, it may have been impossible to estimate true changes of the biotype during follow-up.

Nevertheless, the findings of the regression analysis corroborate the results of previous studies also indicating that at implant sites exhibiting a healthy peri-implant mucosa, a thick tissue biotype was associated with a lower frequency of facial soft-tissue recessions (i.e., MR values) over time when compared with sites exhibiting a thin biotype [6, 20].

In conclusion and within its limitations, the present study has indicated that simultaneous soft-tissue grafting using CTG had a beneficial effect on the maintenance of peri-implant health.

## Data Availability

Not applicable.
